# Improvement of anxiety in ADHD following goal-focused cognitive remediation: a randomized controlled trial

**DOI:** 10.3389/fpsyg.2023.1212502

**Published:** 2023-11-17

**Authors:** Kjersti T. Hanssen, Erlend J. Brevik, Milada C. Småstuen, Jan Stubberud

**Affiliations:** ^1^District Psychiatric Center Nedre Romerike, Division of Mental Health Services, Akershus University Hospital, Lørenskog, Norway; ^2^Faculty of Health Science, Oslo Metropolitan University, Oslo, Norway; ^3^Department of Psychology, University of Oslo, Oslo, Norway; ^4^Department of Research, Lovisenberg Diaconal Hospital, Oslo, Norway

**Keywords:** ADHD, executive function, anxiety, depression, goal management training, goal attainment scaling

## Abstract

**Introduction:**

Despite the high prevalence and detrimental consequences of cognitive and executive dysfunction in ADHD, the evidence base of cognitive remediation in the adult ADHD population is sparse. Executive problems can increase both anxiety and depression in ADHD. Thcus, it is important to develop treatment options for adults with ADHD, aiming to improve goal-directed behavior and mood. Goal Management Training (GMT) is an intervention that has received empirical support in improving executive functions and mood in normal aging and for various neurological and psychiatric conditions. The present randomized controlled trial investigated the effects of a goal-focused intervention combining 1) group-based GMT incorporating psychoeducation about ADHD and 2) guidance in implementing individual goals for coping with executive problems in everyday life, compared to treatment as usual (TAU). The primary outcome was perceived executive functioning in everyday life. Secondary outcomes included psychological well-being (anxiety, depression, and coping with ADHD symptoms).

**Methods:**

We recruited 81 adult participants with a verified ADHD diagnosis (*M*_age_   = 31 years). Inclusion was based upon the presence of executive functioning complaints. The participants were randomly assigned to either the intervention or TAU. The intervention group (*n* = 41) received 16 hours of GMT and psychoeducation, in addition to 4 individual sessions focusing on formulating goals. The goals were assessed in 6 bi-weekly phone calls in the first three months following the group sessions. Participants in the TAU group (*n* = 40) received standard, individually-adapted follow-up in an outpatient psychiatric health care setting. All participants were assessed at baseline, post-intervention, and at 8-month follow-up (main measurement time point).

**Results:**

Significant improvements in everyday executive functioning, psychological wellbeing, and symptoms of ADHD from baseline to 8-month follow-up were reported in both groups. The intervention group reported a significantly higher reduction in symptoms of anxiety compared to TAU. Conclusions. Our findings provide support for considering cognitive remediation as a treatment option for patients with ADHD.

**Clinical Trial Registration**: https://clinicaltrials.gov/study/NCT04638283?term=NCT04638283&rank=1, identifier: NCT04638283.

## Introduction

The evidence base for cognitive remediation in the adult attention deficit hyperactivity disorder ADHD population is sparse (Kim et al., 2018). This is surprising given the high prevalence and possible detrimental consequences of executive and cognitive problems (Barkley, 1997; Hervey et al., 2004; Seidman et al., 2004; Sergeant, 2005; Willcutt et al., 2005).

ADHD, which is referred to as Hyperkinetic disorder in the International Classifications of Diseases (ICD-10), is characterized by a persistent and impairing pattern of inattention, hyperactivity, and impulsivity (World Health Organization, 1992). The condition is among the most common neuropsychiatric disorders, known to persist into adulthood for a considerable proportion of patients (Castells et al., 2018). Indeed, prevalence estimates of approximately 3–5% in children (Giacobini et al., 2018) and 1.4–3.6% in adults (Kooij et al., 2019) are commonly reported. As ADHD can persist as a chronic, life-long condition despite optimal treatment, there is a need for evidence-based treatments focusing on symptom management (Caye et al., 2019) and reducing comorbid psychiatric disorders (Katzman et al., 2017). Importantly, executive functions are likely to influence emotional symptoms in ADHD, and have, along with emotional symptoms, been reported to mediate the relationship between ADHD and quality of life (Zhang et al., 2021).

Executive functions can be defined as those *abilities necessary to formulate goals, carry them out effectively* (Lezak et al., 2004, p. 35), and enable a person to *engage successfully in independent, purposive, self-serving behavior* (Lezak et al., 2012, p. 37). Executive problems can compromise goal-directed behavior and utilization of personal resources by making it difficult to allocate attentional resources, suppress inappropriate responses, and keep goals in mind (Barkley, 2010). Being among the core symptoms of ADHD, executive problems can partly explain why the condition causes significant economic burdens for society (Sayal et al., 2018), in addition to negative consequences for the individuals affected. ADHD has indeed been associated with poorer education and occupational functioning (Halmøy et al., 2009; Barkley, 2010; Fredriksen et al., 2014), antisocial acts, marital difficulties, and lower socioeconomic status (Faraone et al., 2015; De Crescenzo et al., 2017), as well as negative self-esteem (Matsuura et al., 2009; Torrente et al., 2014).

The management of executive control may have implications for aspects of emotional health, and the capacity for emotional self-regulation (Rudea et al., 2004). This is indeed important in the ADHD population, where the prevalence of comorbid psychiatric disorders is high (McGough et al., 2005; Torgersen et al., 2013; Anker et al., 2019), with anxiety (Prevatt et al., 2015) and depression (Nelson and Liebel, 2018) being the most common. A large Norwegian population-based study (Solberg et al., 2018) reported that adults with ADHD have a four to nine times higher prevalence of psychiatric conditions compared to the remaining population. For anxiety, these ADHD patients reported prevalence rates as high as 26% for women and 18% for men. For depression, the prevalence rates were even higher (28% for women and 20% for men) (Solberg et al., 2019). Executive dysfunction has indeed been reported to increase both anxiety and depression in ADHD (Knouse et al., 2013; Han et al., 2016; Torrente et al., 2017).

The relationships between ADHD, executive functions, and psychological well-being are, however, complex. Firstly, as described in ICD-10 (World Health Organization, 1992), symptoms of ADHD, anxiety, and depression overlap, making both assessment and evaluation of treatment effects challenging. Secondly, treatment of one of the conditions can interfere with other conditions. Of note, in line with the US Food and Drug Administration warnings, stimulants can increase anxiety (Reimherr et al., 2017). On the other hand, several studies (Mattos et al., 2013; Bloch et al., 2017) have found that methylphenidate can reduce anxiety (Reimherr et al., 2017). Thirdly, symptoms of anxiety and depression can occur as a consequence of executive problems, e.g., shortcomings in completing tasks and meeting deadlines, and not as symptoms of mood disorders *per se* (Mohamed et al., 2020). For college students, self-reported executive problems have indeed been associated with anxiety, beyond the relationship with ADHD symptoms (Jarrett, 2016). For university students with ADHD, weak organization and planning have been shown to predict mood symptoms (Mohamed et al., 2020). Based on findings from a recent study, where executive dysfunction was found to mediate the association between ADHD symptoms and anxiety in adolescents, the authors pinpointed executive dysfunction as an important treatment target in alleviating anxiety (Haugan et al., 2022).

Since the etiology of ADHD is understood as multifactorial and the symptoms are understood as context-dependent (Faraone et al., 2015), most treatment guidelines incorporate behavioral interventions (Graham et al., 2011) and multimodal treatment approaches have been recommended (Swanson et al., 2017; Kooij et al., 2019). Still, pharmacological treatment is often the first choice, and often the only treatment offered. Of note, in a Norwegian survey, only 20% of adults with ADHD reported that they had been offered treatment options other than medication (Solberg et al., 2019). Pharmacological ADHD treatment alone, however, is insufficient due to several reasons: (1) Despite having been in clinical use for more than eight decades (Bradley, 1937), the quality of its evidence has been rated as low, particularly for long-term effects (Hinshaw and Arnold, 2015; Storebø et al., 2015). (2) Approximately 30% of ADHD patients do not respond to or do not tolerate psychostimulants (Bouffard et al., 2003; Chou et al., 2012). (3) Treatment discontinuation is common (Gajria et al., 2014), and even for those responding, compliance is often reduced (Graham et al., 2011; Castells et al., 2018), due to adverse effects (Gajria et al., 2014) such as sleeping problems and decreased appetite (Storebø et al., 2015), and arrested height development has also been reported (Swanson et al., 2017). (4) As pharmacological treatments only improve aspects of the ADHD-symptoms, additional follow-up, such as psychoeducation or guidance in implementing beneficial routines, is usually required (Hinshaw and Arnold, 2015). Moreover, in addition to the insufficiency of pharmacological treatment, misuse of prescription stimulants has been described as a serious problem (Weyandt et al., 2016).

The complexity and mixed etiology identifying the condition may partly explain the lack of evidence for pharmacological treatment alone (Leahy, 2018). In line with this, non-pharmacological interventions, offered in addition to pharmacological treatment, have been reported to increase satisfaction with health care (Solberg et al., 2019). However, the evidence for non-pharmacological interventions for adults with ADHD is sparse (De Crescenzo et al., 2017). Previous studies have reported beneficial effects of cardio exercise for executive functions, attention, and behavior (Den Heijer et al., 2017; Lambez et al., 2020). A recent meta-analysis of the effects of non-pharmacological interventions on cognitive symptoms in ADHD (Lambez et al., 2020) highlighted the positive effects of psychological interventions, including cognitive behavioral therapy (CBT), neuro/biofeedback, and cognitive training. Lambez et al. (2020) conclude that behavioral interventions can be effective when the goal is to improve cognitive and executive symptoms in ADHD.

Goal Management Training (GMT) (Levine et al., 2011) is an intervention that specifically targets executive functions, aiming to enhance goal-directed behavior. In GMT, the participants train to increase awareness of errors and strategies while facing complex challenges. They learn to stop ongoing behavior, define goal hierarchies, adjust goals, and monitor goal attainment. GMT draws upon theories regarding sustained attention, goal processing, and mindfulness (Kabat-Zinn, 2012). The intervention relies on metacognitive strategies, including sustained attention and alerting techniques, to reengage endogenous attention processes. Importantly, in GMT, generalization to everyday life is heavily emphasized (Levine et al., 2011).

Beneficial effects of GMT have been reported for normal aging (Levine et al., 2007), neurological conditions (Krasny-Pacini et al., 2014; Stubberud et al., 2014; Tornås et al., 2016; Richard et al., 2019), and for psychiatric conditions such as depression (Stubberud et al., 2021) and schizophrenia/psychosis risk syndromes (Haugen et al., 2022). Furthermore, GMT has been found to be effective when integrated with other methods (Krasny-Pacini et al., 2014).

For adults with ADHD, the evidence base for GMT is sparse. To our knowledge, only one previous randomized controlled trial (RCT) (In de Braek et al., 2017) has investigated the effects of GMT in this population. The results were described as promising, but the number of participants was small (*n* = 27). Furthermore, the intervention was modified and combined with psychoeducation. Thus, the results must be interpreted with caution. One recent study (Jensen et al., 2021) found that GMT was associated with improvements in core executive functions such as inhibitory control and self-regulation in everyday life for adults with ADHD. Furthermore, they reported improvements in the orienting network postulated by the attention network theory (Petersen and Posner, 2012). The authors concluded that GMT might be a potential mechanism of change for adults with ADHD. However, as the study followed a self-control design, the authors (Jensen et al., 2021) underlined the possibility that practice effects may have contributed to the improvements. Further studies including control conditions were called for.

The present RCT investigated the effects of a goal-focused intervention combining (1) group-based GMT incorporating psychoeducation about ADHD and (2) guidance in implementing individual goals for coping with executive problems in everyday life. The main aim was to compare participation in the intervention to treatment as usual (TAU). The main outcome was perceived executive functioning in everyday life. Secondary outcomes were psychological well-being (anxiety, depression, and coping with symptoms related to ADHD). Furthermore, we expected that participants in the GMT group would be able to formulate and implement individual GAS goals for coping with executive problems in everyday life, and that goal attainment would be sustained throughout the three-month-long implementation phase.

## Materials and methods

The study was approved by the Regional Committees for Medical and Health Research Ethics, Norway (2019/81), conducted in accordance with the Helsinki Declaration, and reported according to CONSORT criteria (Schulz et al., 2010). The study was also preregistered at ClinicalTrials.gov with the identifier NCT04638283. The study followed the design of a parallel-group RCT.

### Participants and procedures

We recruited 81 participants with a verified diagnosis of ADHD, currently receiving follow-up for their ADHD at District Psychiatric Center Nedre Romerike at Akershus University Hospital in the inclusion period lasting from June 2019 until April 2021, based on the following eligibility criteria, that were similar for both groups:

- Diagnosis of ADHD, operationalized as Hyperkinetic disorder, described in ICD-10 as F90.0, F90.1, F90.8, or F90.9. Thirty-seven of the included participants had been diagnosed with ADHD prior to being referred to the clinic, and 44 had recently been diagnosed with ADHD at the clinic, but prior to being included in the study
- Age between 18 and 60
- Subjective complaints about executive problems affecting everyday life, operationalized as a score of 60 or above on BRIEF-A, GEC (described below), or as reported in the inclusion interview (e.g., starting too many tasks at the same time, resulting in problems with completing projects, or hyper-focusing too long on one task at the expense of other tasks)
- Motivation to work on executive problems to increase coping in everyday life. Before inclusion, all potential participants were asked to consider their motivation, and no one was advised to participate unless willing to spend the time and energy required for participation
- Adequate language skills to participate in group discussions
- No central nervous system injury or disease
- No ongoing substance abuse
- No psychopathology that would negatively interfere with participation in the intervention, e.g., ongoing psychosis, acute suicidal risk, or personality disorders too severe to be handled in a group-based out-patient setting
- Participation in the study did not put any limitations on pharmacological treatment options. 66/80 (81.5%) were receiving pharmacological treatment for their ADHD while participating in the study

As part of the public, specialized healthcare system, the District Psychiatric Center Nedre Romerike offers assessment and treatment related to a range of psychiatric conditions, including ADHD. Being among the largest in Norway, the clinic served an adult population of approximately 125,000 during the inclusion epoch for this trial. Thus, based on the prevalence of 2.8% (Kooij et al., 2019), an estimated 3,500 adults diagnosed with ADHD lived in the area served by the clinic during the inclusion period.

**Figure 1:**
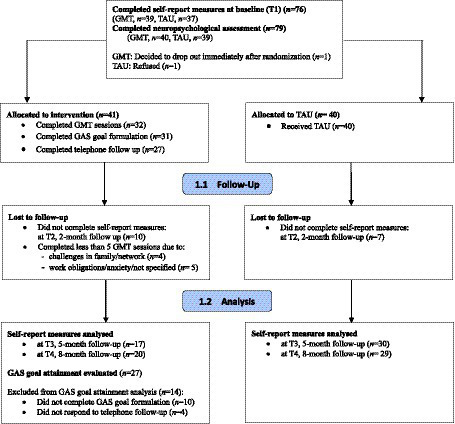
Study flow chart.

First, we offered brief information about the study to patients potentially fulfilling the eligibility criteria. Next, we offered patients choosing to learn more about the study written information and opportunities to ask questions. Volunteers fulfilling the inclusion criteria provided written informed consent and were randomized in a 1:1 ratio to the intervention group or the control group by a person independent from the study team. See Figure 1 for the study flow chart.

### The intervention

The intervention consisted of: (1) Group-based GMT and (2) Individual guidance in formulating GAS goals for coping with executive problems in everyday life.

#### Group-based GMT

Group-based GMT consisted of eight, weekly scheduled psychoeducational sessions, in accordance with the GMT protocol (Stubberud et al., 2013; Tornås et al., 2016), each lasting 2 × 45 min in addition to a 15 min long break. We made some minor adjustments by compressing the original nine GMT sessions into eight and by addressing ADHD-related topics when relevant. The topics presented during the eight sessions are presented in Table 1. We applied the Norwegian version of the manual and workbook (Stubberud et al., 2013).

**Table 1:** Topics in GMT group sessions.

Session		Topics
1	Absent mind. Present mind.	Absent-and present-mindedness; relations to failures and goal-attainment. Mental laboratory. Mindfulness (body-scan exercise).
2	Absentminded slip-ups.	Factors increasing the risk of slip-ups. Relation of absentmindedness to other capacities. Mindfulness (breathing exercise).
3	Automatic pilot.	Following routines can increase the risk of absentminded slip-ups.
4	Stop the automatic pilot.	Introduce/practice a strategy of stopping to increase present-mindedness and monitor current behavior.
5	Mental blackboard.	Checking as a metaphor for working memory. Consequences of the limited capacity of “The Mental Blackboard.” Breathing exercise.
6	State your goal.	Importance of explicitly stating goals. Goal loss and reinstatement.
7	Making decisions.	Conflicting goals. To-do-list as a tool for keeping overview of relevant goals and facilitating decision-making.
8	Splitting tasks into subtasks. Checking.	Dividing overwhelming tasks into subtasks. STOP-STATE-SPLIT-technique. Adapting current goals and ongoing behavior in response to environmental changes. Final summary of all sessions.

Assignments between sessions included tasks such as recording absentminded slip-ups and activities that went well, along with present-mindedness practice. The cutoff for completing the GMT intervention was having participated in at least five of the eight sessions.

#### GAS goals

Additionally, during four individual sessions, each lasting 45 min, scheduled biweekly before or after the group sessions, the participants in the intervention group received guidance in formulating individual GAS goals (Kiresuk and Sherman, 1968) for coping with executive problems in everyday life. GAS provides a method for quantifying the attainment of individualized goals. The participants can establish as many GAS goals as desired. As goal attainment can be summarized across different goals and participants, the method is suitable for the evaluation of individual goal attainment on a group level. Following the GAS procedure, thoroughly described by Turner-Stokes (2009), for each individual goal, we operationalized different levels of outcomes on a five-point scale: −2 = goal attainment much less than expected, −1 = a little less than expected, 0 = the expected outcome, +1 a little bit better than expected and + 2 = goal attainment much better than expected. We monitored GAS goal attainment during biweekly telephone calls, each lasting approximately 5 min, in the first 3 months after completion of the GMT group sessions.

### Treatment as usual

TAU consisted of individually adapted follow-up for ADHD from a team comprising psychiatrists, psychologists, social workers, psychiatric nurses, and a psycho-motoric physiotherapist. Participation in the study did not affect pharmacological treatment or access to sessions with the health care professionals in the multi-disciplinary team. The mean numbers of individual sessions provided to the participants in the intervention group in addition to the intervention, and to participants in the control group during the eight-month-long inclusion period are presented in Table 2 in the Results section. Blinding related to group allocation was not possible due to the nature of the intervention.

**Table 2:** Treatment as usual.

	Intervention group (*n* = 39) Mean (SD)	Control group (*n* = 40) Mean (*SD*)	*P*-values	Both groups (*n* = 79) Mean (*SD*) (min-max)
Psychiatrist	2.31 (2.60)	5.30 (4.53)	<0.001	3.82 (3.97) (0–20)
Psychologist	2.59 (5.26)	2.88 (4.45)	n.s.	2.73 (4.84) (0–24)
Social worker	0.72 (2.63)	0.15 (0.43)	n.s.	0.43 (1.88) (0–15)
Psychiatric nurse	0.48 (2.26)	0.10 (0.50)	n.s.	0.29 (1.64) (0–14)
Psycho-motoric physiotherapist	0.08 (0.35)	0.23 (1.42)	n.s.	0.15 (1.03) (0–9)
Total sessions	6.18 (6.81)	8.65 (5.95)	n.s.	7.43 (6.47) (0–28)

Individual sessions. Numbers of individual sessions of up to 45 min, provided by health care providers in the multidisciplinary team during the eight-month long inclusion-period (for the intervention group: provided in addition to the intervention).

### Data collection

At baseline (T1), the participants in both groups underwent comprehensive neuropsychological assessment and responded to self-report measures regarding executive functions, psychological well-being, and intensity of ADHD symptoms. The same self-report measures were given to both groups at T2 (2 months past baseline, the time-point coinciding with completion of the psychoeducational phase), at T3 (5 months past baseline, the time-point coinciding with completion of the telephone follow-up), and finally at T4 (8 months past baseline). We scored GAS goal attainment in the intervention group between T2 and T3.

### Neuropsychological assessment

Baseline neuropsychological assessment included the widely used and well-validated instruments summarized in Table 3.

**Table 3:** Neuropsychological tests.

Cognitive domain	Test
General cognitive ability	Wechsler Adult Intelligence Scale-IV (Wechsler, 2009)
Verbal learning and memory	Rey Auditory Verbal Memory Test (Schmidt, 1996) Logical Memory, Wechsler Memory Scale-Revised (Wechsler, 1987)
Visual memory	Rey Complex Figure Test and Recognition Trial (Meyers and Meyers, 1995)
Working memory	Digit Span from WAIS-IV (Wechsler, 2009) Spatial Span from Wechsler Memory Scale-III (Wechsler, 1997)
Divided attention	Trail Making Test B (Reitan and Wolfson, 1985)
Verbal fluency	The Controlled Oral Word Association Test (Spreen and Strauss, 1998)
Information processing speed	WAIS-IV, Digit-symbol Test S-IV Trail Making Test A (Reitan and Wolfson, 1985)
Attention	D-KEFS Color Word Interference Test (Delis et al., 2001) Digit Vigilance Test (Lewis and Rennick, 1979) Conners CPT-III (Keith Conners et al., 2018)
Motor Speed/Coordination	Grooved Pegboard Test (Klove, 1963)

### Self-report questionnaires

Executive functions were assessed by the Norwegian version of BRIEF-A (Roth and Gioia, 2005; Rabin et al., 2006). It consists of 75 items rated as being a problem never, sometimes, or often in the past 6 months. Higher scores on BRIEF-A indicate higher levels of executive complaints. BRIEF-A provides a Global Executive Composite (GEC) score as well as two index scores: The Behavioral Regulation Index (BRI) comprising the Inhibit, Shift, Emotional Control and Self Monitor subscales, and the Metacognition Index (MI) comprising the Initiate, Working Memory, Plan/Organize, Task monitor and Organization of Materials subscales. BRIEF-A raw scores were converted to T-scores, a normally distributed scale with a mean score of 50 and a *SD* of 10. High reliability of the BRIEF-A has been reported; Cronbach’s alpha of the BRI and MI has been found to be 0.94 and 0.96, respectively (Waid-Ebbs et al., 2012).
Psychological well-being was assessed with the Hopkins Symptom Checklist-25 (HSCL-25) (Derogatis et al., 1974). This screening instrument measures the presence and intensity of symptoms of anxiety (items 1–10) and depression (items 11–25). It has been validated in Norway (Sandanger et al., 1999). The participant rates each item on a scale ranging from 1 (not bothered) to 4 (extremely bothered), indicating the degree to which the behavior described in the item has been a problem in the last week. The sum of item scores on each symptom scale and the total scale are divided by the number of items answered.
Current ADHD symptoms were assessed by the Adult ADHD Self-Report Scale (ASRS-v1.1). This 18-item questionnaire was developed in conjunction with the World Health Organization to assess symptoms of ADHD (Kessler et al., 2005). The patient rates each item on a 5-point scale ranging from “never” to “very often,” indicating the degree to which the feelings and behavior described in the item have been a problem in the last 6 months.

Information about current psychiatric status/comorbidity was collected from each patient’s journal. Individual goal attainment was measured by GAS (Kiresuk and Sherman, 1968), following the standard GAS procedure, thoroughly described by Turner-Stokes (2009).

### Outcomes

The primary outcome was the BRIEF-A GEC score, and the primary time point was at 8 months (T4). Secondary outcomes included the HSCL-25 (total score and sub-scores for anxiety and depression) and the ASRS-v1.1.

### Statistical analyses

The lack of previous studies on GMT in ADHD represents a challenge in estimating the required sample size. However, findings from a recent study (Cameron et al., 2020) examining the use of GMT in individuals with obsessive-compulsive disorder suggest that based on an average effect size of η^2^ *p* = 0.054 for neuropsychological variables observed across all results in Cameron et al.’s study, with a critical alpha = 0.05 and 80% power, a sample size of *n* = 23 per group would be required to reliably detect the smallest desired effects. Based on the above, we would need a total of 46 individuals in our study, but to allow for a dropout rate of about 40%, we aimed to include at least 64 participants.

Statistical analyses were performed using the Statistical Package for the Social Sciences (SPSS) Version 26. Continuous variables were described with mean and standard deviation (*SD*), categorical data as counts and percentages. Crude differences between groups were assessed using t-tests for continuous variables and Chi-square for pairs of categorical variables.

We used a linear mixed model for repeated measures to assess possible between-and within-group differences over time. We applied a 2×4 mixed-design, with Group (GMT, TAU) as the between-subjects factor, and Session (T1: baseline, T2: 2 months, post-GMT, T3: 5 months, post telephone follow-up, and T4: 8 months) as the within-subjects factor. In line with previous recommendations (Løvstad et al., 2016), clinically meaningful change in the BRIEF-A was operationalized as an improvement of one *SD*. All tests were two-sided and *p*-values <0.05 were considered statistically significant. All analyses were considered exploratory so no correction for multiple testing was done.

## Results

### Demographic and clinical characteristics

Demographic and clinical characteristics of the study population at baseline are presented in Table 4. Median age was 30.0 years (range 18–55), and 47 (58%) of the participants were women. The proportion of women in the study population was higher than expected, based on a previously-reported gender ratio of 3:1 males to females in adults with ADHD (Young et al., 2020), although the gender disparity has been reported to disappear in the adult ADHD population (Faraone et al., 2015). Furthermore, 19/81 (23.5%) were diagnosed with anxiety, 23/81 (28.4%) with depression, 4/81 (4.9%) with a personality disorder, and 2/81 (2.5% with Tourette Syndrome). Based on observations/information revealed post inclusion, an additional assessment was considered necessary for some participants, resulting in 3/81 (3.7%) being diagnosed with pervasive developmental disorder (PDD). Comorbidities were comparable between the groups. There were no significant baseline differences between the intervention and control groups regarding age, education, gender, intelligence quotient (WAIS-IV), BRIEF-A composite scores (GEC, MI, BRI), HSCL-25 scores (total, anxiety, depression) or ASRS-v1.1 total score. The proportion of employed individuals/students was significantly higher in the intervention group [25/40 (63%)] compared to the TAU group [14/41 (34%)]. The mean scores on the BRIEF-A were approximately 2–2.5 *SD* above the normative mean for both the intervention group and the control group.

**Table 4:** Demographical and clinical characteristics of the study population at baseline.

	*n* assessed	Intervention group (*n* = 41)	Control group (*n* = 40)	*P*-values
Mean age (range)	41/40	31.2 (18–55)	31.4 (20–49)	n.s.
Years of education (range)	41/40	11.3 (9–16)	11.9 (9–17)	n.s
Female (%)	41/40	20 (48.8%)	27 (67.5%)	n.s
Work/studies (%)	41/40	14 (34.1%)	25 (62.5%)	0.011 (sign)
FSIQ (*SD*)	38/38	104 (9.5)	104 (13.1)	n.s.
GEC (BRIEF-A), *T*-score (*SD*)	37/35	74.8 (8.8)	76.3 (8.3)	n.s.
MI (BRIEF-A), *T*-score (*SD*)	37/35	76.4 (8.9)	77.4 (9.3)	n.s.
BRI (BRIEF-A), *T*-score (*SD*)	37/35	68.4 (8.9)	70.1 (8.8)	n.s.
HSCL-25 total	39/37	2.3 (0.5)	2.4 (0.6)	n.s.
HSCL-25 anxiety	39/37	2.1 (0.4)	2.2 (0.6)	n.s.
HSCL-25 depression	39/37	2.3 (0.7)	2.4 (0.6)	n.s.
ASRS-v1.1	34/35	48.0 (11.5)	51.2 (9.5)	n.s.

FSIQ, Full Scale IQ; GEC (BRIEF-A), General Executive Composite from the Behavior Rating Inventory of Executive Function for Adults; MI, Metacognition Index; BRI, Behavioral Regulation Index; HSCL-25, Hopkins Symptom Checklist-25; ASRS-v1.1, Adult ADHD Self Report Scale version 1.1. BRIEF-A provides *T*-scores, a normally distributed scale with *M* = 50 and a standard deviation (SD) of 10. Higher BRIEF-A scores represent higher levels of executive complaints.

### Neuropsychological characteristics

Neuropsychological characteristics of the study sample are presented in Table 5. On a group level, the study sample achieved *T*-scores within the normal range on all neuropsychological tests.

**Table 5:** Neuropsychological characteristics of the study population.

Test (*n* assessed)	Mean *T*-scores (*SD*) if not otherwise specified		
	Intervention group	TAU group	Total study population
WAIS-IV	53 (8.1)	52 (9.3)	53 (8.7)
Block design (40/39)			
Similarities (39/39)	57 (9.7)	58 (9.9)	58 (9.7)
Digit span (40/39)	50 (7.6)	47 (10.5)	49 (9.2)
Matrix reasoning (39/39)	55 (11.4)	56 (10.0)	55 (10.6)
Vocabulary (40/39)	51 (6.2)	51 (7.3)	51 (6.7)
Arithmetic (38/38)	46 (8.4)	47 (10.0)	47 (9.1)
Symbol search (38/38)	54 (9.9)	55 (11.5)	54 (10.7)
Visual puzzles (38/38)	54 (11.0)	55 (8.8)	55 (9.9)
Information (38/39)	51 (7.8)	49 (9.5)	50 (8.6)
Digit symbol (39/39)	50 (7.9)	50 (9.3)	50 (8.6)
VCI (38/38)	Std. score 106 (10.8)	Std. score 106 (13.5)	Std. score 106 (12.1)
PRI (38/38)	Std. score 107 (14.2)	Std. score 108 (13.5)	Std. score 108 (13.7)
WMI (38/38)	Std. score 96 (11.7)	Std. score 95 (14.1)	Std. score 95 (12.9)
PSI (38/38)	Std. score 104 (13.1)	Std. score 105 (15.6)	Std. score 104 (14.3)
FSIQ (38/38)	Std. score 104 (9.5)	Std. score 104 (13.1)	Std. score 104 (11.3)
TMT A (40/39)	52 (13.9)	53 (14.0)	52 (13.8)
TMT B (39/39)	49 (11.4)	46 (11.8)	47 (11.6)
D-KEFS Stroop Color/Word Time (39/39)/Errors (16/14)	48 (12.4)/47 (14.3)	46 (12.0)/50 (8.6)	47 (12.2)/48 (11.9)
D-KEFS Stroop Switching Time (38/39)/Errors (15/14)	47 (12.8)/46 (13.4)	45 (12.6)/50 (6.0)	46 (12.7)/48 (10.5)
COWAT (38/39)	48 (10.2)	48 (12.2)	48 (11.2)
RAVLT Total Recall (39/39) Immediate Recall (39/38) Delayed Recall (38/38) Recognition Trial (35/31)	49 (14.0) 52 (13.0) 52 (12.4) 49 (11.6)	51 (13.2) 54 (12.8) 54 (12.6) 47 (13.1)	50 (13.5) 53 (12.8) 53 (12.4) 48 (12.3)
Logical memory Immediate Recall (39/37) / Delayed Recall (39/37)	50 (14.2) 51 (10.6)	50 (10.8) 49 (10.2)	50 (12.6) 50 (10.4)
RCFT Immediate Recall (38/38) Delayed Recall (38/38) Recognition Trial (38/38)	48 (12.7) 48 (13.6) 51 (10.8)	50 (14.1) 49 (14.4) 46 (11.8)	49 (13.4) 49 (13.9) 48 (11.6)
DVT Errors (35/33)/ Time (68)	47 (12.6) 45 (9.9)	46 (12.0) 44 (10.2)	46 (12.3) 44 (10.0)
Grooved Pegboard Dominant.hand (38/38) Non-dominant hand. (37/38)	51 (11.6) 50 (11.0)	50 (13.6) 48 (12.3)	51 (12.6) 49 (11.7)
CPT-III (21/26) Detectability	50 (11.6)	52 (11.7)	51 (11.6)
CPT-III Error type (21/26) Omissions/Commissions/Perseverations	50 (13.4) / 51 (8.8) / 51 (12.0)	48 (5.9) / 56 (12.3) / 51 (9.7)	49 (9.9) / 53 (11.0) 51 (10.7)
CPT-III RTS (21/26) HRT/HRT SD/ Variability HRT Block Change HRT ISI Change	52 (11.7) / 49 (15.1) 49 (13.7) 56 (8.2) 48 (8.2)	47 (9.1) / 47 (9.6) 50 (11.0) 53 (6.3) 50 (9.9)	49 (10.5) / 48 (12.2) 50 (12.1) 54 (7.3) 50 (9.1)

WAIS-IV, Wechsler Adult Intelligence Scale-IV; Std. score, standard score; VCI, verbal comprehension index; PRI, perceptual reasoning index; WMI, working memory index; PSI, processing speed index; FSIQ, full scale intelligence quotient; TMT, trail making test; D-KEFS, Delis-Kaplan executive function system; COWAT, controlled oral word association test; RAVLT, Rey auditory verbal learning test; RCFT, Rey complex figure test and recognition trial; DVT, digit vigilance test; CPT-III, Conners Continuous performance test 3rd edition; RTS, reaction time statistics.

### Treatment as usual

Comparison with independent samples t-tests revealed no significant difference in the total number of TAU sessions provided to the intervention and control groups. However, the number of sessions with a psychiatrist was larger in the control group (mean of 5.30 sessions, as compared to 2.31 sessions in the intervention group, *p <* 0.001).

### Outcome measures

#### Primary outcome

**Figure 2:**
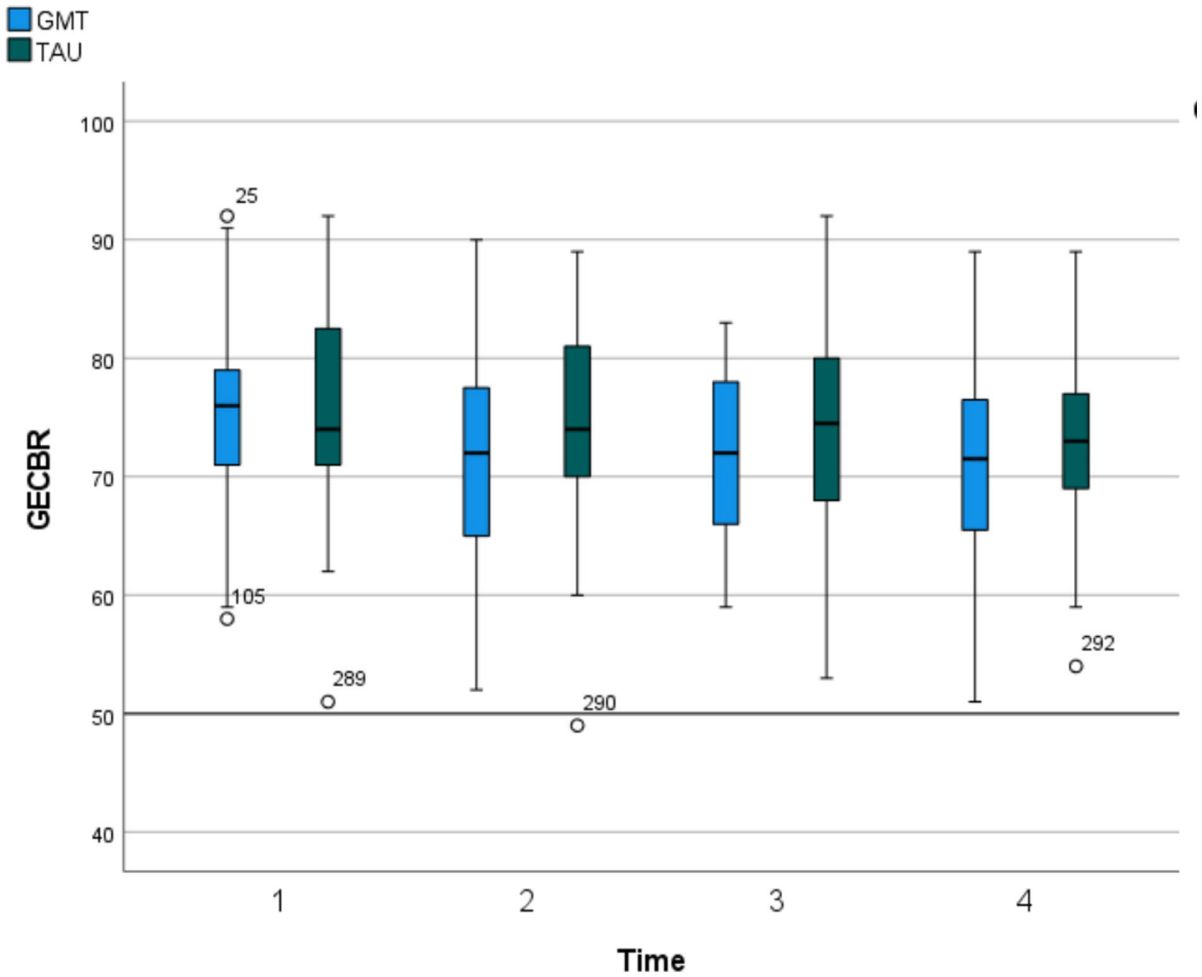
Primary outcome. Global executive composite from behaviour rating inventory of executive function (*T*-scores). GECBR, Global Executive Composite from Behaviour Rating Inventory of Executive Function, Adult. Scores listed are *T* scores (M550, SD510), with higher scores indicating greater impairment. Time = Baseline (T1), 2 months past baseline (T2), 5 months past baseline (T3), 8 months past baseline (T4).

Our data did not reveal any statistically significant difference between the groups on the BRIEF-A GEC at any time point. The trajectory over time was similar in both groups (p for group*time interaction =0.29). The analyses did reveal a significant effect of time (*p* < 0.001), as both groups reported significantly fewer symptoms at T4 compared to T1. Mean scores and 95% confidence intervals for the intervention and control groups on the BRIEF-A GEC at the four measurement points (T1-T4) are presented in Figure 2. On a group level, the mean improvement in the primary outcome measure (BRIEF-A GEC) was approximately 0.5 *SD* in the intervention group (an improvement of 4.78 from 74.71 at T1 to 69.93 at T4), and somewhat less in the control group (an improvement of 2.97 from 76.26 at T1 to 73.29 at T4), thus less than the one *SD*, which previously has been defined as a clinically meaningful change (Løvstad et al., 2016). Ten of the 46 participants (22%) responding to the BRIEF-A GEC at the eight-month follow-up (T4) reported at least 1 *SD* improvement from baseline. Of those improving at least 1 *SD*, 7 were women and 3 were men. Four were in the youngest age group (18–31), and 6 in the oldest age group (32–55).

#### Secondary outcomes

**Figure 3:**
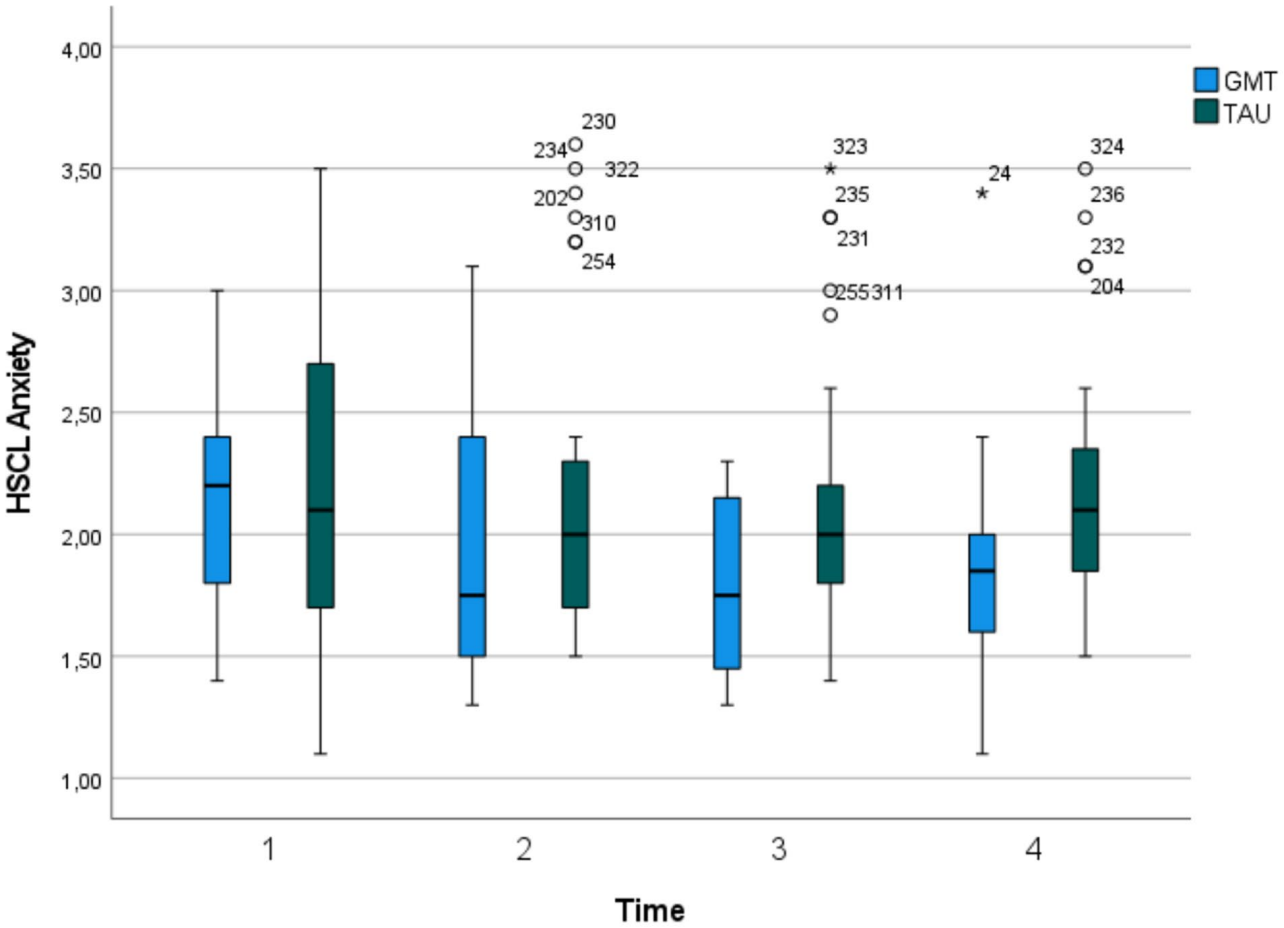
Secondary outcome, HSCL-25 Anxiety subscale. Time = Baseline (T1), 2 months past baseline (T2), 5 months past baseline (T3), 8 months past baseline (T4). HSCL Anxiety, Hopkins Symptom Checklist-25 (HSCL-25) Anxiety subscale.

Comparisons of main and secondary outcome measures are presented in Table 6. The trajectory over time was different for the intervention group compared to the control group, with the intervention group improving significantly more over time on items reflecting anxiety symptoms. Significant time-by-group interactions were found for both the anxiety subscale and the total score of HSCL-25. Mean scores and 95% confidence intervals for the intervention and control groups on the HSCL-25 anxiety subscale at the four measurement points (T1–T4) are presented in Figure 3.

**Table 6:** Comparisons of main and secondary outcome measures.

Mean scores for main and secondary outcomes	*n* assessed GMT/TAU	GMT T1-T4	TAU T1-T4	Group-time interactions (*p*)	Time (*p*)
BRIEF-A, GEC	20/29	74.71–69.93	76.26–73.29	n.s. (0.290)	<0.001
BRIEF-A, BRI	20/29	68.39–62.87	70.20–67.76	n.s. (0.421)	0.004
BRIEF-A, MI	20/29	76.30–70.96	77.23–74.44	n.s. (0.273)	<0.001
HSCL-25, anxiety	22/31	2.14–1.82	2.18–2.21	0.014	0.011
HSCL-25, depression	22/31	2.30–2.15	2.40–2.46	n.s. (0.329)	0.025
HSCL-25, total	22/31	2.29–1.97	2.35–2.35	0.048	0.004
ASRS-v1.1	21/27	48.09–43.39	51.43–47.59	n.s. (0.454)	0.004

GMT, goal management training; TAU, treatment as usual; BRIEF-A, behavior rating inventory of executive function for adults; GEC, global executive composite score; BRI, behavioral regulation index; MI, metacognition index; HSCL-25, Hopkins symptom checklist-25; ASRS-v1.1, adult ADHD self report scale version 1.1.

On HSCL-25, a cut-off of clinical mental distress of 1.75 has been suggested (Skogen et al., 2017). A total of 12 participants scored below this cut-off at T4 (8 in the intervention group and 4 in the control group). Of the participants scoring below cut off 4/12 were women, 4/12 were in the youngest age group (18–31 years) and 8/12 were in the oldest age group (32–55 years).

As presented in Table 6, both groups improved significantly from baseline on all secondary measures (all BRIEF-A index scores, HSCL-25 total and subscale scores and ASRS.v-1.1).

### Completion and dropout-rates

In the intervention group, 32 of the 41 participants attended at least five of the eight GMT sessions, which was set as the cut-off for being a GMT completer. This indicates a GMT dropout rate of 22%. One participant received a summary of the two last sessions individually, administered by video link due to changes in their job situation which made it impossible to attend in person. Those specifying reasons for not attending mentioned illness or critical events in their family or close network, work obligations, and anxiety.

Thirty-one of the 41 participants in the intervention group completed GAS goal formulation (representing a dropout rate of 24%). The participant who received the last group sessions by video-link also completed GAS goal formulation during the video-link sessions. Twenty-seven of the 31 participants (87%) completing GAS goal formulation responded to the bi-weekly telephone follow-up and were assessed for goal attainment, administered between T2 and T3.

Regarding self-report measures, the response rate varied considerably between the two groups. Whereas 30 participants (75%) in the control group responded at the five-month follow-up (T3) and 29 (73%) responded at the eight-month follow-up (T4), only 17 (42%) and 20 (49%) participants in the intervention group responded at 5 and 8 months, respectively.

### GMT: completers vs. dropouts

A comparison of participants completing and dropping out of the GMT intervention is presented in Table 7. Chi-square tests revealed no significant differences between the dropouts and completers regarding age, gender, years of education, employment/student status, comorbid psychiatric disorders, or receiving pharmacological ADHD treatment during the study period. However, independent sample *t*-tests revealed a significantly higher score on the WAIS-IV Perceptual Reasoning Index (PRI) in the GMT completing group, also resulting in a higher WAIS-IV total score (FSIQ) in this group. There were no significant differences between completers and dropouts on the other WAIS-IV composite scores (Verbal Comprehension Scale, Working Memory Scale, or Processing Speed Scale).

**Table 7:** GMT-completers vs. dropouts.

*n =* 41	GMT-completers Mean (*SD*) if not otherwise specified	GMT-dropouts *M* (*SD*) if not otherwise specified	*P*
Age (*SD*)	31.78 (8.73), *n* = 32	28.89 (7.39), *n* = 9	n.s.
Female/Male	*n* = 15/17	*n* = 5/4	n.s.
Years of education (*SD*)	11.25 (2.19), *n* = 32	11.67 (2.35), *n* = 9	n.s.
Being a student or employed (yes/no)	*n* = 11/21	*n* = 3/6	n.s.
WAIS-IV FSIQ	105.3 (9.39), *n* = 30	97.88 (8.08), *n* = 8	0.049
WAIS-IV VCI	106.60 (11.25), *n* = 30	105.13 (9.22), *n* = 8	n.s.
WAIS-IV PRI	109.87 (13.63), *n* = 30	97.13 (12.07), *n* = 8	0.022
WAIS-IV WMI	97.30 (11.99), *n* = 30	91.88 (*SD* 10.01), *n* = 8	n.s.
WAIS-IV PSI	105.07 (*SD* 13.35), *n* = 30	97.38 (*SD* 10.54), *n* = 8	n.s.
Comorbid depression (yes/no)	*n* = 10/22	*n* = 0/9	n.s.
Comorbid anxiety (yes/no)	*n* = 7/25	*n* = 2/7	n.s.
Comorbid developmental disorder (yes/no)	*n* = 1/31	*n* = 1/8	n.s.
Comorbid Tourette Syndrome (yes/no)	*n* = 1/31	*n* = 0/9	n.s.
Comorbid personality disorder (yes/no)	*n* = 1/31	*n* = 1/8	n.s.
ADHD-medication during study-epoch (yes/no)	*n* = 25/7	*n* = 6/2	n.s.

WAIS-IV, Wechsler adult intelligence scale-fourth edition; FSIQ, full scale intelligence quotient (IQ); VCI, verbal comprehension index; PRI, perceptual reasoning index; WMI, working memory index; PSI, processing speed scale.

### Measurement of goal attainment

Thirty-one participants in the intervention group (76%) succeeded in formulating individual GAS goals for coping with executive problems in everyday life. They set between one and three goals (*M* = 1.65), and altogether 51 goals were set. Twenty-seven of the 31 participants initially formulating goals (87%) responded to at least five of the six phone calls and were defined as completers of the GAS intervention. Goal attainment was scored following the procedure described by Turner-Stokes (2009), where a *T*-score of 50 represents goal attainment as expected, 1 *SD* is 10, and higher scores represent better performance. On a group level, an improvement of approximately two *SD*s occurred from baseline until phone-call number six (at T3). The mean GAS score at baseline was 31.4 (*SD* = 3.5), improving to 50.48 (*SD* = 14.8), representing a mean GAS change score of 20.7 (*SD* = 12. 8). The participants decided the number and contents of the goals themselves. The goals could be classified into four different categories:

Planning and organizing (22 goals), e.g.: “Getting out of bed before nine o’clock 5 days per week,” “Eating at least three meals a day 3 days per week” or “Completing an efficient study session of at least 30 min, seven times per week.”
Practicing/utilizing GMT strategies in everyday life (12 goals), e.g.: “Practicing the STOP-technique during dedicated sessions of 30 min duration four times per week” or “Practicing mindfulness five times per week.”
Physical activity (11 goals), e.g.: “Going for a run three times per week,” or “Completing a set of at least 15 push-ups, sit-ups, and squats three times per week.”
Pleasure-activities (6 goals), e.g.: “Listening to music four times a week,” or “Spending time with friends one time per week.”

## Discussion

The current RCT investigated the effects of an intervention combining GMT and individual goal setting, aiming to improve executive functioning and psychological well-being for adults with ADHD and executive complaints. Treatment effects were compared to TAU, comprising individually-adapted follow-up for ADHD from a multidisciplinary team in a psychiatric outpatient setting. The main outcome was self-reported executive functioning. Secondary outcomes were anxiety, depression, and the intensity of ADHD symptoms. We expected that participants in the intervention group would successfully formulate and implement individual GAS goals for coping with executive problems in everyday life, and that goal attainment would sustain throughout the three-month-long implementation phase.

Significant improvements from baseline to 8 months were seen for all the outcome measures in both groups. Thus, our results indicate that both a goal-focused, non-pharmacological treatment approach, and standard follow-up in an outpatient psychiatric clinic, are beneficial for psychological well-being and coping with ADHD and executive problems in everyday life.

### Primary outcome

The intervention did not demonstrate any additional effect in reducing perceived executive dysfunction. However, evaluation of the effects of multi-faceted interventions is challenging, and several factors may have contributed to the lack of significant differences in improvements between the groups.

Both interventions included components that could have facilitated the identification of situations where exerting executive control was required, resulting in the application of remaining reserves in executive functions. Of note, subjective executive complaints were among the inclusion criteria, and all participants had the opportunity to address these in the individual sessions. Accordingly, the differences between the intervention and TAU turned out to be less than originally planned. Future studies investigating the separate effects of interventions in multidisciplinary programs are warranted. Such studies will be particularly relevant in the ADHD population, given the typically complex and multifaceted nature of challenges pertaining to the condition.

In addition to TAU, both groups received a comprehensive neuropsychological assessment that included feedback. On a group level, all neuropsychological test scores at baseline fell within the normal range. Awareness of individual strengths and resources may have enhanced therapeutic processes and contributed to the significant improvements in mood observed in both groups. This is in line with previous studies reporting benefits of neuropsychological assessment with feedback upon self-esteem (Bennett-Levy et al., 1994), insight (Watt and Crowe, 2018), acceptance (Gruters et al., 2021), adherence to treatment and symptomatic distress (Fallows and Hilsabeck, 2013).

Of note, mean neuropsychological test scores within the normal range illustrate clearly that there is no evidence in our study of an “ADHD-specific neuropsychological profile.” Importantly, the high scores on the self-report measures of executive problems nevertheless indicate that executive problems are indeed present in this population, underscoring the importance of a broad assessment approach, not solely based on neuropsychological test scores. It is well-recognized that neuropsychological test scores are suboptimal measures of executive functions due to the high level of structure characterizing the test situations (Garcia-Molina et al., 2012).

This trial was not designed to compare pharmacological and non-pharmacological treatment effects. As we made no restrictions regarding pharmacological ADHD treatment, participants in both groups had equal options for sessions with a psychiatrist, where they could discuss whether such treatment should be initiated, terminated, or optimized. Of note, registration of the TAU content revealed a significantly higher number of sessions with a psychiatrist provided to the control group. A possible explanation for this difference can be that the less time-consuming control condition left those participants with more time and energy to focus on pharmacological issues. Simultaneously, the participants in the intervention group may have prioritized the intervention over psychiatrist sessions, either because they felt it was sufficient to meet their needs, or because it was too demanding to simultaneously focus on pharmacological issues. Further studies investigating the separate effects of pharmacological and non-pharmacological treatments in ADHD are called for.

Our findings are in line with findings from a previous RCT (Hagen et al., 2020), where patients with major depression and self-reported executive deficits took part in either GMT or computerized cognitive training. As in the current trial, improvements in executive functioning from baseline occurred in both groups, but analyses did not reveal any significant group differences in improvement (Hagen et al., 2020). Notably, as the improvements were not sustained until the two-year follow-up, the authors concluded that improvements in everyday executive functions may require maintenance or additional treatment (Hagen and Stubberud, 2021).

### Secondary outcomes

Analyses did reveal significant improvements in anxiety as an effect of the intervention. We consider this an important finding, providing support for a broad treatment approach in psychiatric health care, where interventions from the cognitive remediation field can complement traditional psychotherapeutic interventions, even when the aim is to improve mood. Given the considerable overlap and complex relationships between symptoms of mood disorders, ADHD, and executive problems, this makes considerable sense. Furthermore, this finding provides support for considering executive dysfunction as an important treatment target when the goal is to alleviate anxiety (Haugan et al., 2022).

The anxiety finding is particularly relevant for the approximately 30% of ADHD patients who do not respond to or tolerate psychostimulants (Bouffard et al., 2003). The finding is in line with previous findings that anxiety can improve as a result of increased attentional control/improved self-regulation (Rudea et al., 2004) and improved organization skills (Jarrett, 2016; Mohamed et al., 2020). Treatment of anxiety in the ADHD population is particularly important, as anxiety has been reported to increase the ADHD-symptom load (Reimherr et al., 2017).

GMT comprises several components that have previously been reported to reduce anxiety, including implementing strategies for executive coping in everyday life (Jarrett, 2016; Mohamed et al., 2020), mindfulness (Kabat-Zinn, 2012; Hofmann and Gómez, 2017), and the general therapeutic effects associated with being part of a group, meeting others and receiving psychoeducation (Reimherr et al., 2017).

Statistically significant reductions in self-report measures do not necessarily imply clinical significance. Moreover, it can be discussed how meaningful the significant reductions measured by the HSCL-25 were for functioning in everyday life. On a group level, the intervention group’s anxiety subscale mean score reduced from 2.14 to 1.82. On the total scale, it reduced from 2.29 to 1.97. For the HSCL-25, total scores of 1.75 and above have been suggested as an indication of clinical mental distress (Skogen et al., 2017). Cut-off scores of 1.67 for men and 1.75 for women have also been suggested (Sandanger et al., 1998). Of the 22 participants in the intervention group responding to the HSCL-25 at the eight-month follow-up, a total of 8 (37%) scored below 1.75. Thus, despite the statistically significant reductions, the scores on HSCL-25 remained within the clinical range for approximately two-thirds of the intervention participants.

The factor structure of the HSCL-25 has also been questioned, and a unidimensional model has been described as more appropriate (Skogen et al., 2017). Furthermore, it has been argued that patients do not necessarily discriminate among symptoms; improvement in one area can be experienced as improvements in other areas (Reimherr et al., 2017). On the other hand, significantly better improvements in the intervention group relative to the control group did not occur on the measures of executive functioning (BRIEF-A) or intensity of ADHD symptoms (ASRS-v1.1), indicating that the effects of the intervention were more specifically related to anxiety.

### Goal attainment

As we expected, at least a proportion of the participants in the intervention group [31/41 (76%)] successfully formulated GAS goals for coping with executive problems. Furthermore, the majority [27/31 (87%)] responded to the telephone follow-up. On a group level, an improvement of about two *SD*s occurred from baseline and up to the level perceived as expected and valued by the participants. The participants decided the content of their individual goals themselves. The goals could qualitatively be classified into the following four categories: Planning and organizing (22 goals), Practicing/utilizing GMT strategies in everyday life (12 goals), Physical activity (11 goals), and Pleasure activities (6 goals). The first two categories clearly relate to executive functioning, underlining the importance of executive coping for the participants.

The improvements in both executive coping and anxiety in the current study are in line with previous research reporting a reduction in anxiety as a consequence of improvement in executive coping (Jarrett, 2016; Mohamed et al., 2020). Our findings are also in line with previous suggestions, that anxiety in ADHD may arise when cognitive processing abilities are overwhelmed by the demands of the environment (Schachar et al., 1995; Haugan et al., 2022), and that emotional symptoms in ADHD may be caused by problems related to coping with daily life (Zhang et al., 2021). Improvement of anxiety as a result of guidance in individual goal setting related to activity has also been reported in older veterans (Gould et al., 2021).

The completion rates were considerably higher for the GAS goal intervention compared to the GMT intervention. One participant even completed the GAS goal intervention despite dropping out of the GMT intervention. These findings may indicate that the GAS intervention is more feasible for adults with ADHD, compared to the more time-consuming GMT intervention.

The satisfactory completion rates and successful GAS goal attainment in the current trial are in line with a previous, similar trial (Hanssen et al., 2016), concluding that GAS was a feasible and robust method in cognitive rehabilitation for patients with multiple sclerosis (MS) and cognitive/executive complaints. Beneficial results of GAS have also been reported in various other settings, including special education, rehabilitation, general medical health programs, pain management, and substance abuse treatment (Malec, 1999). GAS has, for a long time been a well-established method within the rehabilitation field (Turner-Stokes, 2009). Our results indicate that the method can apply within a psychiatric setting as well.

### Strengths and limitations

To our best knowledge, this was the first RCT investigating the effects of an intervention combining group-based GMT (Levine et al., 2011) and individual GAS goal-setting (Kiresuk and Sherman, 1968), aiming to help adults with ADHD cope with executive problems in everyday life. Strengths of the study included the use of a sound methodological design, data collection in a naturalistic setting in one of the largest outpatient clinics in Norway, and inclusion only of patients with a verified ADHD diagnosis. The baseline assessment revealed average general mental ability scores in the study sample. The findings can thus be generalized to adults seeking help for coping with problems related to ADHD in a psychiatric outpatient setting. It must be noted that the study only recruited participants with adequate language skills to participate in group discussions and that patients with central nervous system injury, ongoing substance abuse, or psychopathology too severe to be handled in a group-based outpatient setting were excluded. Registration of comorbidity in the study population revealed high rates of comorbid disorders (e.g., depression, anxiety), which is common in the ADHD population (McGough et al., 2005; Torgersen et al., 2013; Prevatt et al., 2015; Nelson and Liebel, 2018; Anker et al., 2019). Thus, our findings may also reflect effects not directly related to ADHD but to concurrent mental disorders. Furthermore, as inclusion was voluntary, we were only able to recruit participants willing to try out a novel treatment approach. Blinding was not possible due to the nature of the intervention. Consequently, we cannot rule out the possibility that awareness of group allocation and the purpose of the study may have affected the responses.

As participation in the study did not affect access to sessions with the health care professionals in the multi-disciplinary team, and no alternative or scam condition was provided to the control group, the total of sessions differed between the groups. This represents a methodological flaw, as the possibility that improvements could be related to other factors than the intervention (e.g., receiving attention from health care providers) cannot be ruled out.

A considerable limitation was the high attrition rates. Only 78% of the intervention group completed the main part of the intervention, GMT. Notably, similar dropout rates were reported in a recent exploratory study of GMT for patients with ADHD (Jensen et al., 2021). Comparisons between GMT completers and dropouts in the current trial did not reveal any statistically significant differences regarding age, gender, employment/student status, receiving pharmacological ADHD treatment during their study participation, or comorbid psychiatric disorders. Attrition in the current study was particularly high in the intervention group at the time points for answering self-report questionnaires at 5 and 8 months. Because of the high attrition rates in our study, there is limited power to detect small differences between the groups, increasing the chance of type-II errors.

The data does not provide the basis for determining why the response rates for the self-report measures were considerably higher in the control group. One hypothesis could be that participation in the intervention was energy-consuming, thus leaving those participants with less energy to complete and return the self-report questionnaires. This is in line with findings from a previous qualitative study, where adults with ADHD were interviewed about their experiences of participating in group-based GMT (Nordby et al., 2021). Even though most participants experienced beneficial effects, such as expanded perspectives, increased personal growth, and coping with ADHD in everyday life, some participants described taking part in the intervention as burdensome. Another possibility is that higher attrition could relate to the significantly lower proportion of occupational or academic participation in the intervention group. We cannot rule out the possibility that unknown variables might have contributed to both attrition and employment status.

One possible explanation for attrition in both groups could be that paying attention to the study was perceived as more interesting at the beginning and that completing paperwork later on was less motivating. Furthermore, we believe that the use of paper-and-pencil versions of the questionnaires was a major source of attrition. Participants in both groups frequently reported that they had lost the questionnaires or forgotten to return them. This observation lends support for digital data collection in future research.

The fact that the study was partially undertaken during the COVID-19 pandemic had implications for both intervention delivery and the everyday life of participants. In accordance with social distancing rules, we had to reduce the size of some of the groups from six to four to keep appropriate distance within the available rooms. The consequences of participants dropping out of group sessions were more noticeable in smaller groups, providing the remaining participants with fewer discussion partners and thus fewer options for shared experiences. Even more importantly, social distancing rules and other pandemic restrictions may have influenced executive coping and psychological well-being for all participants. The psychological impacts of quarantine have indeed been described as substantial and wide-ranging (Brooks et al., 2020). Yet, as inclusion and randomization were undertaken continuously, the effects of the pandemic did not differ systematically between the intervention and control groups.

## Conclusion

The results from the current trial indicate that both TAU (individually-adapted, multi-disciplinary follow-up for ADHD in an outpatient psychiatric setting) and a goal-focused approach (combining group-based GMT with GAS goal-setting) can be beneficial for improving everyday executive functioning, psychological well-being, and symptoms of ADHD. Significantly larger improvements in anxiety were seen in the intervention group compared to the TAU group, suggesting that executive dysfunction might be considered a key target when treating mood problems for patients with ADHD.

## Data availability statement

The datasets presented in this article are not readily available because of restrictions specified in the study consent-form, and conditions for approval from the local ethics committee, concerning patient confidentiality and participant privacy. Requests to access the datasets should be directed to JS, jan.stubberud@psykologi.uio.no.

## Ethics statement

The studies involving humans were approved by The Regional Committees for Medical and Health Research Ethics, Norway (2019/81). The studies were conducted in accordance with the local legislation and institutional requirements. The participants provided their written informed consent to participate in this study.

## Author contributions

KH contributed to the planning and conducting of the study, administering the intervention, and writing the manuscript. EB contributed to the inclusion of participants, administration of the intervention, and reviewing of the manuscript. MS contributed to statistical analyses and reviewed the manuscript. JS contributed to the planning and conducting of the study and reviewing the manuscript. All authors contributed to the article and approved the submitted version.
