# Supplementary material for: Improvement of anxiety in ADHD following goal-focused cognitive remediation: a randomized controlled trial
Source: Front Psychol. 2023 Nov 17;14:1212502. doi: 10.3389/fpsyg.2023.1212502 (PMC10690829; doi:10.3389/fpsyg.2023.1212502)
Supplement: Supplementary file 1 [file Data_Sheet_2.PDF]

Supplementary Material

Supplementary Table 1. GMT-Completers vs Dropouts

n=41	**GMT-completers**<br>Mean (*SD*)<br>if not otherwise specified	**GMT-dropouts**<br>*M* (*SD*)<br>if not otherwise specified	***P***
Age (*SD*)	31.78 (8.73), *n*=32	28.89 (7.39), *n*=9	n.s.
Female/Male	*n*=15/17<br>%= 36.6/41.5	*n*=5/4<br>%=12.2/9.8	n.s.
Years of education (*SD*)	11.25 (2.19), *n*=32	11.67 (2.35), *n*=9	n.s.
Being a student/employed (yes/no)	*n*=11/21<br>%=26.8/51.2	*n*=3/6<br>%=7.3/14.6	n.s.
WAIS-IV FSIQ	105.3 (9.39), *n*=30	97.88 (8.08), *n*=8	.049
WAIS-IV VCI	106.60 (11.25), *n*=30	105.13 (9.22), *n*=8	n.s.
WAIS-IV PRI	109.87 (13.63), *n*=30	97.13 (12.07), *n*=8	.022
WAIS-IV WMI	97.30 (11.99), *n*=30	91.88 (*SD* 10.01), *n*=8	n.s.
WAIS-IV PSI	105.07 (*SD* 13.35), *n*=30	97.38 (*SD* 10.54), *n*=8	n.s.
Comorbid depression (yes/no)	*n*=10/22<br>%=24.4/53.7	*n*=0/9<br>%=0/22	n.s.
Comorbid anxiety (yes/no)	*n*=7/25<br>%=17.1/61	*n*=2/7<br>%=4.9/17.1	n.s.
Comorbid developmental disorder (yes/no)	*n*=1/31<br>%=2.4/75.6	*n*=1/8<br>%=2.4/19.5	n.s.
Comorbid Tourette Syndrome (yes/no)	*n*=1/31<br>%=2.4/75.6	*n*=0/9<br>%=0/22	n.s.
Comorbid personality disorder (yes/no)	*n*=1/31<br>%= 2.4/75.6	*n*=1/8<br>%=2.4/19.5	n.s

ADHD-medication during study-epoch (yes/no)	*n*=25/7<br>%=62.5/17.5	*n*=6/2<br>%=15/5	n.s.

WAIS-IV= Wechsler Adult Intelligence Scale- Fourth Edition, FSIQ= Full scale intelligence quotient (IQ), VCI= Verbal Comprehension Index, PRI= Perceptual Reasoning Index, WMI= Working Memory Index, PSI= Processing Speed Scale.
